# The Dutch version of the Spinal Appearance Questionnaire for adolescents with idiopathic scoliosis: patient-based cross-cultural adaptation and measurement properties evaluation

**DOI:** 10.1007/s43390-023-00746-2

**Published:** 2023-08-10

**Authors:** Dineke G. van de Fliert, Tom P. C. Schlösser, Diederik H. R. Kempen, Joost P. H. J. Rutges, Pepijn Bisseling, Marinus de Kleuver, Miranda L. van Hooff

**Affiliations:** 1 Department of Orthopedic Surgery, Radboud University Medical Center, Geert Grooteplein-Zuid 10, NL, 6525 GA Nijmegen, The Netherlands; 2 Department Orthopedic Surgery, University Medical Center Utrecht, Utrecht, The Netherlands; 3 Department of Orthopedic Surgery, OLVG Amsterdam, Amsterdam, The Netherlands; 4 Department of Orthopaedics and Sports Medicine, Erasmus MC, Rotterdam, The Netherlands; 5 Department of Orthopedic Surgery, Sint Maartenskliniek, Nijmegen, The Netherlands; 6 Department of Research, Sint Maartenskliniek, Nijmegen, The Netherlands

**Keywords:** Scoliosis, Spinal Appearance Questionnaire (SAQ), Physical appearance, Reliability, Validity, Patient-reported outcome measure

## Abstract

**Purpose:**

Adolescent idiopathic scoliosis (AIS) affects the appearance of spine and trunk. The Spinal Appearance Questionnaire (SAQ) assesses the perception of appearance in AIS patients. The aim of this study is to translate and culturally adapt the recommended short version of the SAQ into Dutch and to test its measurement properties.

**Methods:**

A Dutch SAQ (14-item; appearance and expectations domains) was developed following guidelines for translation and cross-cultural adaptation. The COSMIN Study Design checklist was used for measurement properties evaluation. In this multicenter study, the Dutch SAQ, SRS-22R and NPRS (back pain) were administered to 113 AIS patients (aged 15.4 years [SD 2.2], 21.2% male). Floor and ceiling effects were evaluated for content analysis. For reliability, internal consistency (Cronbach’s alpha) and test–retest reliability (ICC; *n* = 34) were evaluated. Predefined hypotheses of relationships with other questionnaires and between subgroups based on scoliosis severity (radiological and clinical) were tested for construct validity. Exploratory factor analysis (EFA) was performed to investigate the validity of the underlying structure of this 14-item questionnaire.

**Results:**

No floor and ceiling effects were found for domains and total scores. Cronbach’s alpha ranged from 0.84 to 0.89. ICCs varied from 0.76 to 0.77. For construct validity, 89% (8/9) of the predefined hypotheses were confirmed. Significant higher scores for the appearance domain were found for subgroups based on radiological (Cobb angle; > 25.0°) and clinical outcomes. (Angle of Trunk Rotation; > 9.0°). A two-factor structure was found (EV 5.13; 36.63% explained variance).

**Conclusion:**

The Dutch SAQ is an adequate, valid and reliable instrument to evaluate patients’ perception of appearance in AIS.

**Level of evidence:**

Level I—diagnostic studies.

**Supplementary Information:**

The online version contains supplementary material available at 10.1007/s43390-023-00746-2.

## Introduction

Adolescent idiopathic scoliosis (AIS) is a three-dimensional deformity of the spine and trunk, resulting in body asymmetry such as uneven shoulder level or rib hump. The deformity mostly arises in otherwise healthy, adolescent girls during the pubertal growth spurt [1]. The patients’ subjective perception of their appearance is often affected by their deformity and can influence the health-related quality of life [2].

In AIS patients, the revised Scoliosis Research Society 22-item (SRS-22r) [3] questionnaire is recommended as a condition-specific instrument to measure quality of life. The SRS-22r includes five questions regarding the patients’ appearance (self-image domain) and gives a broad overview of the general perception of appearance. The Spinal Appearance Questionnaire (SAQ) was developed to provide more detailed information about the patients’ perception of their trunk characteristics [4]. It uses illustrations of physical appearances based on the Walter Reed Visual Assessment Scale. After evaluating the measurement properties, Carreon et al.[5] recommended refining their version of the SAQ to a 14-item questionnaire based on two domains (appearance and expectations) with less burden for the AIS patient. This two-domain version has been translated and cross-cultural adapted into multiple versions of the SAQ and their measurement properties were evaluated [5–9]. Since no validated Dutch version of the SAQ exists, this study aimed to translate and cross-culturally adapt the recommended short English version of the SAQ into Dutch and to evaluate its measurement properties in AIS patients in The Netherlands.

## Methods

The guidelines for cross-cultural adaptation of Beaton et al. [10] were used to translate and adapt the appearance and expectations domain of the SAQ (14 items; recommended short English version [5]) and the instructions into Dutch. The COnsensus-based Standards for the selection of health Measurement INstruments (COSMIN) Study Design checklist for PROMs [11] and the quality criteria of Terwee et al. [12] were used to assess the methodological quality and measurements properties of the Dutch SAQ. The study was approved by the institution’s internal investigational review board. Exemption for ethical approval was obtained by the medical ethical committee of the Radboud University Medical Center (file number: 2021–13280). Informed consent was obtained from all patients and/or parents or caregivers.

### Translation, cross-cultural adaptation and pretest

The multistep approach of Beaton et al. [10], consisting of translation, synthesis, back translation, expert committee review and pretesting, was used (Appendix 1). The expert committee developed the prefinal version of the Dutch SAQ. This version was tested by 30 consecutive AIS patients in three hospitals in the Netherlands. The expert committee discussed the patients’ feedback on the prefinal version and made a few adjustments to the questionnaire (Appendix 1). The patients’ impossibility to evaluate their physical appearance from all sides, including from behind and while bending forward, could not be solved during this phase. After the adjustments, the final version of the Dutch SAQ (Appendix 2) was determined and tested for its measurement properties.

### Study plan to evaluate the measurement properties

From December 2021 to October 2022, a cross-sectional study was conducted involving 113 AIS patients from four hospitals in the Netherlands. The sample size was based on the COSMIN checklist (i.e., ≥ 100 participants and seven times the number of items (14 items)) [11]. AIS patients ranging from 10 to 21 years, with all treatment types (observation, brace treatment and surgery), and who were able to read Dutch were included. Diagnosed psychiatric disorders were an exclusion criterion, due to possible interference with perception of appearance. Study data was obtained through surveys (patient and physician) using Castor EDC (Electronic Data Capture)[13]. Baseline characteristics included age, gender, curve type (according to Lenke classification), coronal Cobb angle, Angle of trunk rotation (ATR; measured with scoliometer during forward bending test) and Risser stage. Measurements were performed on the most recent radiograph by physicians. Thirty-four patients completed the Dutch SAQ twice within a two week interval, for test–retest reliability.

### Self-report measures

The patient survey consisted of Dutch versions of the SAQ, SRS-22R [14] and Numeric Pain Rate Scale (NPRS; back pain). Scoring was performed according to the guidelines of the questionnaires. All patients completed the questionnaires without missing items. The SAQ consists of two domains: appearance (10 pictorial items) and expectations (4 items). The five pictorial answer options of the appearance domain show varying severities of trunk and spinal deformities, scored from 1 (best) to 5 (worst). An exemption is item 9 (position of the head), where score 1 shows backward head position and 5 shows forward head position. The expectations domain is scored from ‘not true’(1) to ‘very true’(5). Scores for the appearance and expectations domain range from 10 to 50 and from 4 to 20, respectively. The total score consisted of the sum of both domains.

The SRS-22r is a condition-specific quality of life measure consisting of 22 items divided into five domains: function, pain, self-image, mental health, and satisfaction with management [14]. Each domain consists of five items scored from 1 (worst) to 5 (best), except for satisfaction with management which has two items. The average domain scores (total domain score divided by the number of items) range from 1 to 5, with higher scores indicating better patient outcomes. The 11-point NPRS (0–10) was used to assess back pain intensity.

### Statistical analysis

Study data are presented as percentages for categorical variables, means with standard deviations for normally and median and ranges for non-normally distributed continuous data. Distribution of the study variables was assessed by the Kolmogorov–Smirnov test. Missing data of baseline characteristics were, if present, clearly described. All statistical analyses were performed in SPSS (IBM® SPSS® Statistics version 27) and a p-value < 0.05 was considered statistically significant.

### Measurement properties

#### Floor and ceiling effects

Floor and ceiling effects of the SAQ were assessed for domain and total scores, and were considered present if > 15% of patients achieved the lowest or highest score, respectively [12].

#### Internal consistency

For each domain and the total score of the SAQ, a Cronbach’s α was calculated. A Cronbach’s α between 0.70 and 0.95 is considered as an acceptable value [12].

#### Reliability and measurement error

Reproducibility of the SAQ was assessed by test–retest reliability analysis of the first and second SAQ with Intraclass correlation coefficients (ICC, two-way random effects, single measurement, for absolute agreement). An ICC ≥ 0.70 demonstrates good test–retest reliability [12]. The standard error of measurement (SEM) and smallest detectable change (SDC) were calculated based on test–retest score differences, providing valuable information on the instrument’s reliability by indicating the range of the theoretical ‘true’ values. The standard deviation of this difference (SD_difference_) was used to calculate the SEM (Formula 1) [15]. The individual SDC (SDC_ind_) and the SDC for a group (SDC_group_) were calculated according Formula 2 and 3, respectively [15].

1 $$SEM= \frac{{SD}_{difference}}{\sqrt{2}}$$

2 $${SDC}_{ind}=1.96\cdot \sqrt{2} \mathrm{SEM}$$

3 $${SDC}_{group}=\frac{{SDC}_{ind}}{\sqrt{n}}$$

#### Hypotheses testing for construct validity

To analyze the construct validity, nine hypotheses were predefined, based on results of previous literature, if applicable, and expected differences between subgroups (Table 1). No previous usable correlations were found for hypothesis 6 and 7. A moderate positive correlation was expected between appearance domain (SAQ) and Angle of trunk rotation (hypothesis 6, Table 1), due to 3D-deformity of the spine and trunk in AIS. In general, AIS does not cause back pain and thus a weak positive correlation was expected between appearance domain (SAQ) and NPRS (back pain) scores (hypothesis 7, Table 1). Subgroups for hypothesis 6 and 7 were based on the median score of radiological (Cobb angle) or clinical (ATR) outcomes to create subgroups with adequate number of patients (> 50 patients [11]). To determine construct validity, ≥ 75% of the results should be in correspondence with the predefined hypotheses (≥ 7; Table 1) [12]. The strength of the correlations is interpreted as “weak” (r = 0.10–0.30), “moderate” (r = 0.31–0.50), or “strong” (r = 0.51–1.00) [16].

**Table 1:** Nine predefined hypotheses for construct validity analysis and the calculated correlations based on the predefined hypotheses

Predefined hypotheses	Correlations in literature	Calculated correlations	Confirmation of hypotheses
Moderate to strong negative correlation between:			
1. Appearance domain (SAQ) and Self-image domain (SRS-22r)	Moderate to strong negative (*r* = − 0.39 to− 0.67) [6–9]	Strong negative (*r* = − 0.55)^a^	Yes
2. Expectations domain (SAQ) and Self-image domain (SRS-22r)	Moderate to strong negative (*r* = − 0.30 to− 0.60) [6–9]	Strong negative (*r* = − 0.53)^a^	Yes
3. Total score (SAQ) and Total score (SRS-22r)	Moderate to strong negative (*r* = − 0.30 to− 0.56) [7–9]	Moderate negative (*r* = − 0.48)^a^	Yes
Weak to moderate negative correlation between:			
4. Appearance domain (SAQ) and pain domain (SRS-22r)	Weak to moderate negative (*r* = − 0.1 to− 0.49) [6–9]	Moderate negative (*r* = − 0.34)^a^	Yes
Strong positive correlation between:			
5. Appearance domain (SAQ) and Cobb angle	Strong positive (*r* = 0.55) [6]	Strong positive (*r* = 0.58)^a^	Yes
Moderate positive correlation between:			
6. Appearance domain (SAQ) and Angle of trunk rotation	n.a	Moderate positive (*r* = 0.50)^a^	Yes
Weak positive correlation between:			
7. Appearance domain (SAQ) and NPRS (back pain)	n.a	Moderate positive (*r* = 0.32)^a^	No
Predefined hypotheses		*P*-value	Confirmation of hypotheses
Significant higher score on Appearance domain (SAQ) for:			
8. Patients with a Cobb angle > 25.0 degrees compared with patients with a Cobb angle ≤ 25.0 degrees		*P* = < 0.001* *Z* = − 4.837	Yes
9. Patients with an ATR > 9.0 degrees compared with patients with an ATR ≤ 9.0 degrees		*P* = < 0.001* *Z* = − 4.480	Yes
Percentage confirmed hypotheses:			88.9%

*SAQ* spinal appearance questionnaire, *SRS*-22r revised Scoliosis Research Society 22-items, *NPRS* numeric pain rate scale, *n* number, *ATR* Angle of Trunk Rotation

^a^Spearman’s rho

^*^p-value < 0.05 was considered significant

#### Structural validity

Exploratory factor analysis (EFA) with principal axial technique and oblique rotation (direct oblimin) was conducted to determine whether the items of the SAQ were covered by one or more factor(s) [12]. In the absence of a clear factor structure, additional EFA with two factors was performed, based on the two factor (two domain) loading previously found [5]. Data factorability was assessed by inspection of inter-item correlations and by calculation of the significance level of the Bartlett test of sphericity and Kaiser–Meyer–Olkin (KMO) measure of sampling adequacy. A KMO value < 0.6 was considered inadequate [17]. Determination of the factors was based on eigenvalues (EV) > 1, factor interpretability, a scree plot, and unique variances of > 5%. Items with factor loadings < 0.4 and cross-loadings of a smaller difference than 0.2 to previous loading were removed, because of inadequate discrimination.

## Results

### Patient characteristics (Table 2)

**Table 2:** Patient characteristics (*n* = 113)

Variables	Values (*n* = 113)
Age (years)	15.4 (2.2)^a^ (ranging 10.1 –21.1)
Gender	
Female	89 (78.8%)^c^
Male	24 (21.2%)^c^
BMI (kg/m^2^)	19.4 (13.0–37.9)^b^
Curve type	
Lenke 1	58 (51.3%)^c^
Lenke 2	11 (9.7%)^c^
Lenke 3	9 (8.0%)^c^
Lenke 4	0 (0.0%)^c^
Lenke 5	31 (27.4%)^c^
Lenke 6	4 (3.6%)^c^
Major Cobb angle (degrees)	25.0 (10.0–68.0)^b^
Maximum Angle of trunk rotation (degrees on scoliometer)	9.0 (1.0–23.0)^b^
Risser stadium	
Risser 0	12 (10.6%)^c^
Risser 1	11 (9.7%)^c^
Risser 2	4 (3.5%)^c^
Risser 3	10 (8.9%)^c^
Risser 4	33 (29.2%)^c^
Risser 5	42 (37.2%)^c^
Unknown	1 (0.9%)^c^
Treatment type	
Observation	45 (39.8%)^c^
Brace	29 (25.7%)^c^
Under brace treatment	20 (17.7%)^c^
Finished brace treatment	9 (8.0%)^c^
Surgery	39 (34.5%)^c^
Waiting list	15 (13.3%)^c^
Post-surgery	24 (21.2%)^c^

*n* number, *BMI* body mass index, kg kilogram, m^2^ square meter

^a^Mean (SD)

^b^Median (range)

^c^Number (%)

Of the 113 included patients, 24 (21.2%) were male. The mean age of the patients was 15.4 years (SD 2.2, range 10–21 years). The median major Cobb angle was 25.0° (range 10.0°- 68.0°). Fifty-eight patients (51.3%) had a Lenke 1 curve type.

### Measurement properties

#### Floor and ceiling effects (Table 3)

**Table 3:** Domain and total scores of the self-report measures and floor and ceiling effects and internal consistency of the Dutch SAQ domains and total score (*n* = 113)

Questionnaire and domain or total scores	Outcomes (*n* = 113)
SAQ (item scored from 1 (best) to 5 (worst))	
Appearance (10 items)	20.0 (11.0–33.0)^b^
Floor effects^e^	0.0 (0.0%)^c^
Ceiling effects^f^	0.0 (0.0%)^c^
Internal consistency	0.84^d^
Expectations (4 items)	12.0 (4.0–20.0)^b^
Floor effects^e^	11.0 (9.7%)^c^
Ceiling effects^f^	16.0 (14.2%)^c^
Internal consistency	0.89
Total score (14 items)	35.0 (15.0–48.0)^b^
Floor effects^e^	0.0 (0.0%)^c^
Ceiling effects^f^	0.0 (0.0%)^c^
Internal consistency	0.85^d^
SRS-22r (item scored from 1 (worst) to 5 (best))	
Function (average of 5 items)	4.6 (2.8–5.0)^b^
Pain (average of 5 items)	4.2 (1.4–5.0)^b^
Self-image (average of 5 items)	3.8 (2.2–5.0)^b^
Mental health (average of 5 items)	3.8 (1.4–5.0)^b^
Satisfaction with management (average of 2 items)	4.5 (1.0–5.0)^b^
Total score (average of 22 items)	4.0 (0.5)^a^
NPRS (item scored from 0 (best)–10 (worst))	
Back pain (1 item)	1.0 (0.0–9.0)^b^

*SAQ* spinal appearance questionnaire, *SRS*-22r revised Scoliosis Research Society 22-items, *NPRS* numeric pain rate scale, *n* number

^a^Mean (SD)

^b^Median (range)

^c^Number (%)

^d^Cronbach’s alpha

^e^Percentage of patients that answered the lowest possible score (i.e. 10 for appearance domain, 4 for expectations domain and 14 for total score)

^f^Percentage of patients that answered the highest possible score (i.e. 50 for appearance domain, 20 for expectations domain and 70 for total score)

The median values of appearance, expectations domains and total scores of the SAQ were 20.0 (11.0–33.0), 12.0 (4.0–20.0) and 35.0 (15.0–48.0), respectively. No floor and ceiling effects were found for each domain and total score (Table 3).

#### Internal consistency (Table 3)

The Cronbach’s alpha, was 0.84 (appearance), 0.89 (expectations) and 0.85 (total score).

#### Reliability and measurement error (Table 4)

**Table 4:** Reliability of the short Dutch SAQ (test–retest; *n* = 34)

Domain	ICC (95% CI)^a^	SD^b^	SEM^b^	SDC_ind_^b^	SDC_group_^b^
Appearance	0.77 (0.59–0.88)	3.15	2.23	6.18	1.06
Expectations	0.76 (0.57–0.87)	3.63	2.57	7.11	1.22
Total	0.77 (0.59–0.88)	5.82	4.11	11.40	1.96

*SAQ* spinal appearance questionnaire, *ICC* intraclass correlation coefficient, *CI* Confidence interval, *SEM* STANDARD error of measurement, *SDC* smallest detectable change

^a^Two-way random effects, single measurement, for absolute agreement

^b^Based on the difference between test and retest scores

The test–retest reliability ranged from 0.76 (95%CI 0.57–0.87) to 0.77 (95%CI 0.59–0.88). The SEM for the total score was 4.11 and for the domains 2.23 (appearance) and 2.57 (expectations). The total score SDC_ind_ and SDC_group_ were 11.40 and 1.96, respectively. The SDC_ind_ and SDC_group_ for the domains can be found in Table 4.

#### Construct validity (Table 1 and Fig. 1 A and B)

**Fig. 1:**
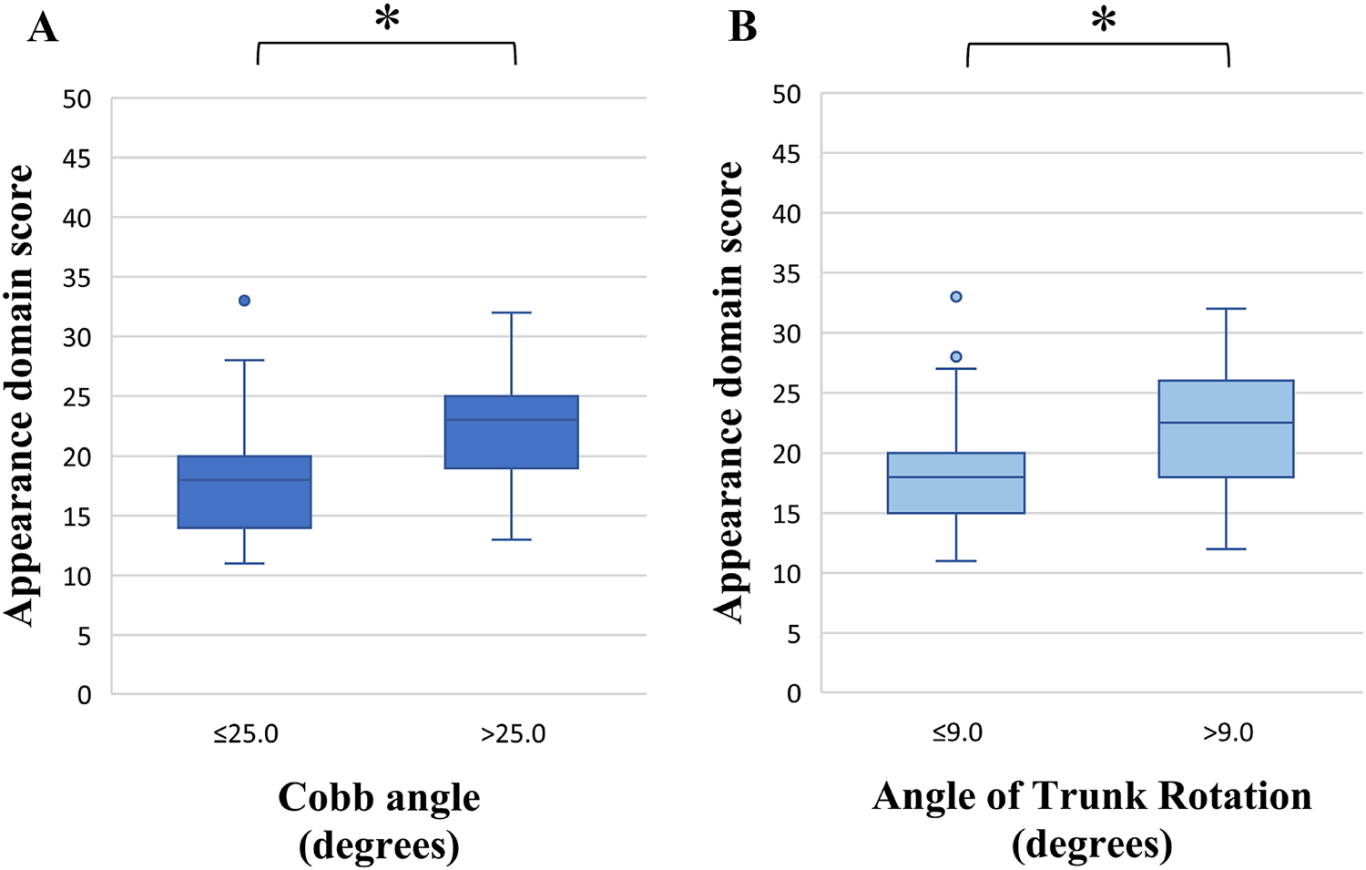
Box-whisker plots for the Appearance domain scores of the SAQ (0–50), with in Fig. 1A) the comparison between two subgroups based on the median score of 25.0° for the Cobb angle (≤ 25.0°; > 25.0°), and in Fig. 1B) the comparison between two subgroups based on the median score of 9.0° for the ATR (≤ 9.0°; > 9.0°). Patients with a Cobb angle of > 25.0° had a significant higher score on the Appearance domain compared with patients with a Cobb angle of ≤ 25.0° (*Z* = − 4.837, *P* =  < 0.001). A significant higher Appearance domain score was found for patients with an ATR of > 9.0° compared with patients with an ATR of ≤ 9.0 (Z = − 4.480, *P* =  < 0.001). *ATR* angle of trunk rotation, *n* number, Q1 first quartile, Q3 third quartile *p < 0.05. **A** Cobb angle (degrees) Cobb ≤ 25.0 (*n* = 58): 14.0 (Q1), 18.0 (Median), 20.0 (Q3) Cobb > 25.0 (*n* = 55): 19.0 (Q1), 23.0 (Median), 25.0 (Q3) **B** Angle of Trunk Rotation (degrees) ATR ≤ 9.0 (*n* = 57): 15.0 (Q1), 18.0 (Median), 20.0 (Q3) ATR > 9.0 (*n* = 56): 18.0 (Q1), 22.5 (Median), 26.0 (Q3)

Eight of the nine (88.9%) predefined hypotheses of the expected correlations were confirmed. Only the hypothesis that the SAQ appearance domain correlated with NPRS for back pain, was not confirmed (r = 0.32, moderate positive correlation).

#### Structural validity (Table 5)

**Table 5:** Characteristics of the Dutch SAQ, including exploratory factor analysis

SAQ item	Mean score (SD)	Floor effect* n* (%)	Ceiling effect* n* (%)	Missing values* n* (%)	Item-total correlation	Item-App correlation	Item-Exp correlation	Factor 1 (App) loading	Factor 2 (Exp) loading
1	2.1 (0.8)	17 (15.0)	1 (0.9)	0 (0.0)	0.54	0.66	0.28	0.63	–
2	1.8 (0.6)	38 (33.6)^a^	0 (0.0)	0 (0.0)	0.52	0.64	0.23	0.65	–
3	1.6 (0.7)	55 (48.7)^a^	0 (0.0)	0 (0.0)	0.42	0.52	0.20	0.44	–
4	2.1 (0.8)	25 (22.1)^a^	1 (0.9)	0 (0.0)	0.73	0.76	0.48	0.66	–
5	2.0 (0.9)	39 (34.5)^a^	1 (0.9)	0 (0.0)	0.58	0.62	0.35	0.45	–
6	2.1 (0.8)	28 (24.9)^a^	0 (0.0)	0 (0.0)	0.62	0.71	0.33	0.63	–
7	2.0 (0.8)	28 (24.9)^a^	1 (0.9)	0 (0.0)	0.52	0.69	0.20	0.70	–
8	2.1 (0.9)	36 (31.9)^a^	0 (0.0)	0 (0.0)	0.51	0.75	0.14	0.85	–
9	2.4 (0.7)	8 (7.1)	1 (0.9)	0 (0.0)	0.34	0.44	0.17	0.25^b^	–
10	1.9 (0.7)	35 (31.0)^a^	0 (0.0)	0 (0.0)	0.58	0.63	0.33	0.55	–
11	3.3 (1.4)	16 (14.2)	32 (28.3)^a^	0 (0.0)	0.82	0.48	0.92	–	0.86
12	3.2 (1.5)	22 (19.5)^a^	30 (26.5)^a^	0 (0.0)	0.72	0.40	0.83	–	0.67
13	2.7 (1.6)	43 (38.1)^a^	27 (23.9)^a^	0 (0.0)	0.63	0.21	0.85	–	0.88
14	2.9 (1.6)	33 (29.2)^a^	28 (24.8)^a^	0 (0.0)	0.73	0.36	0.87	–	0.79
Eigen–value Percentage of variance								5.13	2.27
								36.63	16.24

*SAQ* spinal appearance questionnaire, *SD* standard deviation, *n* number, *App* appearance domain, *Exp* expectations domain

^a^Floor or ceiling effects present (> 15.0%)

^b^Factor loading < 0.4

EFA showed a KMO measure of sampling adequacy value of 0.80 and a significant Bartlett's Test of Sphericity (χ^2^ = 771.07, *df* = 91, *P* < 0.001). Floor effects were found for 11 of the 14 items (19.5% to 48.7%), while ceiling effects were present in the four items of the expectations domain (23.9%–28.3%). The correlations between items score and total score ranged from 0.34 (item 9) to 0.82 (item 11). Item 1 to 10 loaded on factor 1 (EV 5.13; 36.63% explained variance), except for item 9 (factor loading < 0.4). Item 11, 12, 13 and 14 loaded on factor 2 (EV 2.27; 16.24% explained variance). EFA showed two evident factors, explaining 52.87% of the total variance and corresponding to the two factor structure of the original SAQ (appearance [factor 1] and expectations domain [factor 2]).

## Discussion

In this study, the recommended short English version of the SAQ was successfully translated and cross-cultural adapted into Dutch. Its measurement properties are adequate in terms of validity and reliability to assess the appearance of AIS patients in The Netherlands. In this process, the guidelines by Beaton et al. [10] and Terwee et al. [12] and the COSMIN Study Design checklist [11] were followed.

No floor or ceiling effects were found for domain or total score of the Dutch SAQ (Table 3). This reveals that the questionnaire is able to discriminate among the lowest (best) and highest (worst) scores of the domains and the total questionnaire. The SAQ showed acceptable internal consistency, with Cronbach’s α ranging from 0.84 to 0.89, meaning homogeneity of the items. Furthermore, good reliability was found for the test–retest of the SAQ, with ICC’s between 0.76 and 0.77. This shows that the questionnaire is consistent over time in AIS patients. Unlike most previous SAQ translations and as recommended by the COSMIN checklist [11], this study used predefined hypotheses and factor analysis to assess the validity of this 14-item SAQ. Eighty-nine percent (8/9) of the associations (with SRS-22r and NPRS (back pain)) and differences between subgroups based on Cobb angle and ATR) were as expected. Significant higher scores for the appearance domain were found for both radiological (Cobb > 25.0°) and clinical based (ATR > 9.0°) subgroups. This implicates the potential discriminative ability of the appearance domain between patients with a Cobb angle ≤ 25.0° and > 25.0° and between patients with an ATR ≤ 9.0° and > 9.0°. EFA found a two-factor structure of this 14-item SAQ, supporting the previously described two-factor (two-domain: appearance and expectation domain) structure [5]. The two factors explained 53% of the variance, suggesting that other non-measured factors or domains are involved in the concept of patient-experienced appearance.

The calculated internal consistencies of the SAQ are similar to previous translations of this version of the SAQ (appearance domain ranged from 0.89 to 0.94, expectations domain 0.81–0.89, and total scores 0.88–0.91) [5–9]. The Danish, English, German, Spanish and Turkish versions found ICC’s between 0.84 and 0.98 (appearance), 0.67 and 0.97 (expectations), and 0.80 and 0.98 (total score) [5–9].

Although no floor and ceiling effects were found for the domain and total score, both effects were present for almost all items (except for item 1 and 9; Table 5). The discriminative ability of these items might be limited, because of the inability to distinguish within the group of patients scoring the lowest or highest score. In our study, factor analysis showed a factor load < 0.4 for item 9 (position of the head) and removal of this item could be considered. The internal consistency of the appearance domain (factor 1) marginally improved after omission of item 9 (Cronbach’s alpha improved from 0.84 to 0.85). One possible reason might be the non-identical sequence of the illustrations of this item, which may be difficult for patients to recognize this by themselves. Furthermore, the position of the head (sagittal view, item 9) might be more relevant for patients with i.e. kyphosis than with AIS. To our knowledge, no other publication including translations of the SAQ described considerations of omitting item 9. Further research is needed to determine if item 9's effect is unique to the Dutch version. Nevertheless, to maintain questionnaire comparability with other versions, all 14 items are included in the Dutch version.

When considering appearance in patients with AIS, the SRS-22r includes five questions about the patients’ appearance in general (self-image domain). The SAQ includes 10 pictorial questions about scoliosis-specific physical appearances. This study revealed that the SAQ appearance domain strongly correlates with the SRS-22r self-image domain (Table 1, hypothesis 1 [r = 0.55]). Both questionnaires complement each other and provide physicians comprehensive information about patients' appearance perception, which can affect quality of life and treatment outcomes. As such, the SAQ can provide additional detailed information about appearance perception in clinical practice.

Some limitations of this study should be mentioned. First, in this study, we used the appearance and expectations domain of the SAQ for cross-cultural adaptation into Dutch, as recommended by Carreon et al. [5], to reduce patient burden. The original SAQ [4] had 33 items and was subsequently reduced to 20 items with nine domains. Some translations [4, 18–23] used these versions with a different scoring method and thus comparisons with their results were not possible. A second limitation is related to the questionnaire itself, as it measures the patients’ perception of appearance although not every part of their deformity is directly visible to them. Sixty percent of the patients in the prefinal version testing phase were unable to see their own trunk and spine from all directions, indicating difficulties with the patients' perception of appearance. This feedback, along with Simony et al.'s [7]. reported difficulties with two items specific about shoulder and shoulder blade, can be useful for future research whether and how this influences the self-reported values. The third limitation was the limited sample size for subgroup analysis, based on Cobb angle and ATR (hypothesis 6 and 7; Table 1). To obtain an adequate number of patients in each subgroup (> 50 patients [11]), we created subgroups based on median scores. Future research should focus on larger sample size to be able to investigate clinical relevant threshold values for Cobb angle and ATR. Finally, future longitudinal studies are recommended to determine measurement properties as responsiveness and clinically relevant cutoff and change scores to define successfulness of scoliosis treatment.

## Conclusion

The Dutch short SAQ is an adequate, valid and reliable instrument to evaluate the perception of appearance and thereby the health-related quality of life in AIS patients. It is a valuable condition-specific PROM and we recommend it for use in future AIS research to evaluate the outcome of all types of scoliosis treatment in patients with AIS.

### Supplementary Information

Below is the link to the electronic supplementary material.

Supplementary file1 (PDF 84 KB)

Supplementary file2 (PDF 456 KB)

## Data Availability

The data that support the findings of this study are available from the corresponding author, Miranda L. van Hooff, upon reasonable request.

## References

[1] Weinstein SL, Dolan LA, Cheng JC (2008). Adolescent idiopathic scoliosis. Lancet.

[2] Smith PL, Donaldson S, Hedden D (2006). Parents' and patients' perceptions of postoperative appearance in adolescent idiopathic scoliosis. Spine.

[3] Asher MA, Lai SM, Glattes RC (2006). Refinement of the SRS-22 health-related quality of life questionnaire function domain. Spine.

[4] Sanders JO, Harrast JJ, Kuklo TR (2007). The Spinal Appearance Questionnaire: results of reliability, validity, and responsiveness testing in patients with idiopathic scoliosis. Spine.

[5] Carreon LY, Sanders JO, Polly DW (2011). Spinal appearance questionnaire: factor analysis, scoring, reliability, and validity testing. Spine.

[6] Matamalas A, Bago J, D'Agata E (2014). Body image in idiopathic scoliosis: a comparison study of psychometric properties between four patient-reported outcome instruments. Health Qual Life Outcomes.

[7] Simony A, Carreon LY, Hansen KH (2016). Reliability and validity testing of a Danish translated version of spinal appearance questionnaire (SAQ) v 1.1. Spine Deform.

[8] Thielsch MT, Wetterkamp M, Boertz P (2018). Reliability and validity of the Spinal Appearance Questionnaire (SAQ) and the Trunk Appearance Perception Scale (TAPS). J Orthop Surg Res.

[9] Yapar A, Yapar D, Ergisi Y (2021). Reliability and validity of the adapted Turkish version of the spinal appearance questionnaire. Spine Deform.

[10] Beaton DE, Bombardier C, Guillemin F (2000). Guidelines for the process of cross-cultural adaptation of self-report measures. Spine.

[11] Mokkink LB, Prinsen CA, Patrick DL, et al. ( 2023) COSMIN Study Design checklist for Patient-reported outcome measurement instruments. https://www.cosmin.nl/wp-content/uploads/COSMIN-study-designing-checklist_final.pdf# Updated Version July 2019. Accessed march 20

[12] Terwee CB, Bot SD, de Boer MR (2007). Quality criteria were proposed for measurement properties of health status questionnaires. J Clin Epidemiol.

[13] Castor EDC. Castor electronic data capture. https://castoredc.com 27 Aug. 2019, Accessed April 16, 2023,

[14] Schlosser TP, Stadhouder A, Schimmel JJ (2014). Reliability and validity of the adapted Dutch version of the revised Scoliosis Research Society 22-item questionnaire. Spine J.

[15] De Vet HC, Terwee CB, Mokkink LB, et al. ( 2011) Measurement in medicine: a practical guide. Cambridge university press

[16] Cohen J (1992). A power primer. Psychol Bull.

[17] Beavers AS, Lounsbury JW, Richards JK (2013). Practical considerations for using exploratory factor analysis in educational research. Pract Assess Res Eval.

[18] Roy-Beaudry M, Beausejour M, Joncas J (2011). Validation and clinical relevance of a French-Canadian version of the spinal appearance questionnaire in adolescent patients. Spine.

[19] Misterska E, Glowacki M, Harasymczuk J (2011). Assessment of spinal appearance in female patients with adolescent idiopathic scoliosis treated operatively. Med Sci Monit.

[20] Wei X, Zhu X, Bai Y (2012). Development of the Simplified Chinese Version of the Spinal Appearance Questionnaire: cross-cultural adaptation and psychometric properties evaluation. Spine.

[21] Guo J, Lau AH, Chau J (2016). A validation study on the traditional Chinese version of spinal appearance questionnaire for adolescent idiopathic scoliosis. Eur Spine J.

[22] Lee JS, Shin JK, Goh TS (2017). Validation of the Korean version of the Spinal Appearance Questionnaire. J Back Musculoskelet Rehabil.

[23] Babaee T, Moradi V, Rouhani N (2022). Assessment of reliability and validity of the adapted Persian version of the Spinal Appearance Questionnaire in adolescents with idiopathic scoliosis. Spine Deform.

