# Supplementary material for: The Dutch version of the Spinal Appearance Questionnaire for adolescents with idiopathic scoliosis: patient-based cross-cultural adaptation and measurement properties evaluation
Source: Spine Deform. 2023 Aug 10;12(1):79–87. doi: 10.1007/s43390-023-00746-2 (PMC10769903; doi:10.1007/s43390-023-00746-2)
Supplement: Supplementary file 1 — Supplementary file1 (PDF 84 KB) [file 43390_2023_746_MOESM1_ESM.pdf]

Appendix 1: Full report of translation, cross-cultural adaptation and pretest of the short Dutch Spinal Appearance Questionnaire

Translation and cross-cultural adaptation

Primarily, two bilingual translators (Dutch and English, with Dutch as mother tongue) independently translated the short SAQ into Dutch. One of the translators was an orthopedic spine surgeon with clinical expertise in the field of scoliosis. Both Dutch translations were compared and a synthesis was conducted by the translators. Discrepancies were resolved by discussion until consensus was reached. Subsequently, two different and independent, bilingual translators (Dutch and English, with English as native language) performed the back translation from Dutch into English. Both translators, one without medical background, were blinded to the original English SAQ. The expert committee consisted of translators, two orthopedic spine surgeons, a researcher (medical doctor) and methodologist. This committee reviewed all translations and written reports, including the original SAQ. The translations were also examined for semantic, idiomatic and conceptual equivalences. For the expert committee, the meaning of the question 'head chest hips' was unclear in combination with the pictorial items. Therefore, the text was modified into 'De vorm van jouw romp (bovenlichaam)', in English 'The shape of your trunk (upper body)'

Pretest

In total, 30 consecutive patients in three Dutch outpatient clinics tested the prefinal version. Patients who were able to read and speak the Dutch language and with all types of treatment, observation, bracing and surgery, were included. Each patient was asked to complete the questionnaire using the think-aloud method. They were interviewed about the relevance, meaning and understanding of each item, involving text and pictorial items, the response options and potential missing items**.** The comprehension of the instructions and recall period of the questionnaire were also evaluated. The expert committee discussed the feedback of pretesting. Seventeen patients (57%) indicated that they were unable to see their own trunk and spine from all sides and needed support to perceive their actual physical appearance (e.g. mirror, photographs or another person). Furthermore, seven

patients (23%) found it unclear from which direction they looked at the figure in the pictures. Therefore, the views (back, front and side view) were added to the pictorial items of the Dutch version of the SAQ was determined. Twenty-two patients (73%) did not understand what was meant by ‘I want to be more even’ in Dutch. They often asked whether it was about their own body or in comparison with others. To clarify this point, left-right (in Dutch) was added to the question to implicate their own physical appearance. For six patients (20%), it was uncertain for which time point they had to complete the questionnaire (e.g. appearance before surgery or current appearance). Hence, the words ‘at this moment’ were added in Dutch to the instructions of the questionnaire.
