# Supplementary material for: The Dutch version of the Spinal Appearance Questionnaire for adolescents with idiopathic scoliosis: patient-based cross-cultural adaptation and measurement properties evaluation
Source: Spine Deform. 2023 Aug 10;12(1):79–87. doi: 10.1007/s43390-023-00746-2 (PMC10769903; doi:10.1007/s43390-023-00746-2)
Supplement: Supplementary file 2 — Supplementary file2 (PDF 456 KB) [file 43390_2023_746_MOESM2_ESM.pdf]

Appendix 2: The Dutch Spinal Appearance Questionnaire

Vragenlijst over het uiterlijk bij scoliose

Nederlandse versie van de Spinal Appearance Questionnaire (Dutch SAQ)

Gegevens:

Voor- en achternaam: ____________________________

Geboortedatum (dd/mm/jjjj): ______/______/______

Geslacht: ____________________

Datum (dd/mm/jjjj): ______/______/______

Instructies:

Kijk goed naar de volgende afbeeldingen die verschillende vormen van een rug weergeven.
Kleur het bolletje onder de tekening in dat <u>op dit moment</u> het meest op jouw rug lijkt.
Kleur één antwoord in per vraag.

1 **De kromming van jouw rug**
(Kleur één bolletje in)

Van achteren:

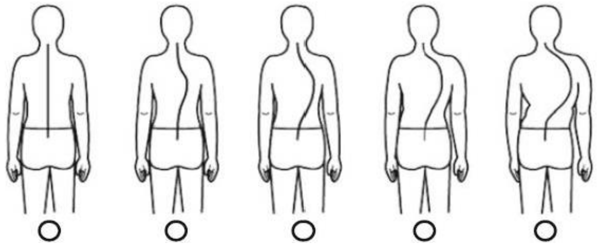

2 **De bolling van jouw ribben**
(Kleur één bolletje in)

Van voren:

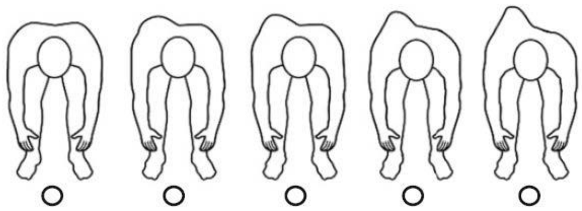

3 **De bolling onderin jouw rug**
(Kleur één bolletje in)

Van achteren:

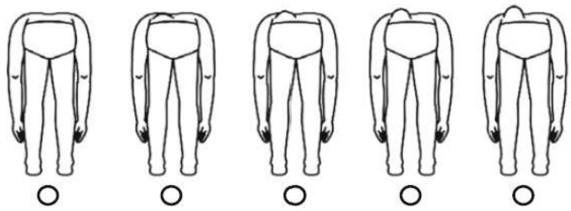

4 **De vorm van jouw romp (bovenlichaam)**
(Kleur één bolletje in)

Van voren:
(of van achteren)

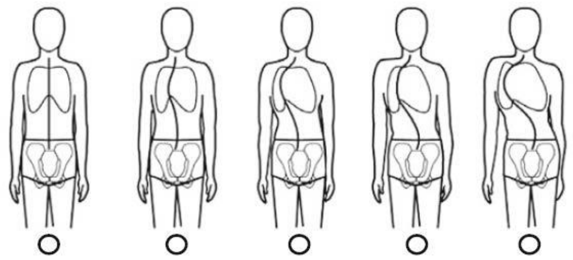

5 **De positie van jouw hoofd ten opzichte van jouw heupen**
(Kleur één bolletje in)

Van voren:
(of van achteren)

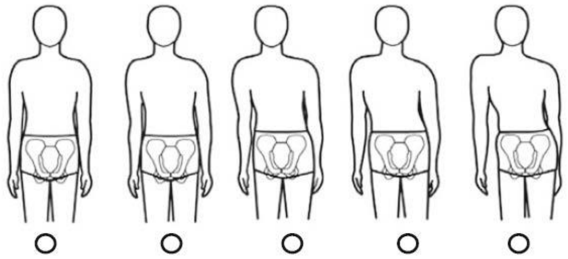

6 **De hoogte van jouw schouders**
(Kleur één bolletje in)

Van achteren:
(of van voren)

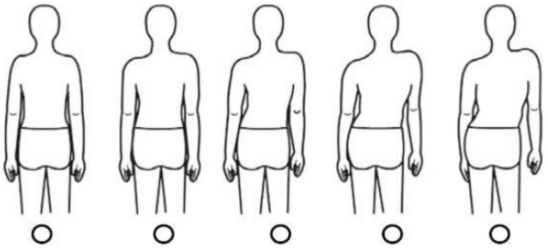

7 **De kanteling van jouw schouderblad**
(Kleur één bolletje in)

Van achteren:

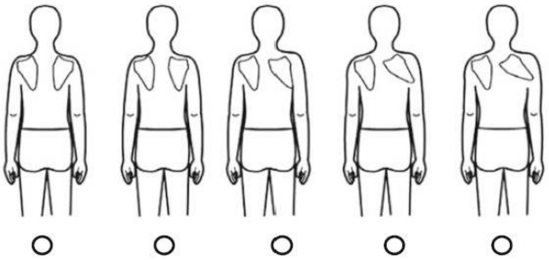

8 **Het uitsteken van jouw schouderblad**
(Kleur één bolletje in)

Van achteren:

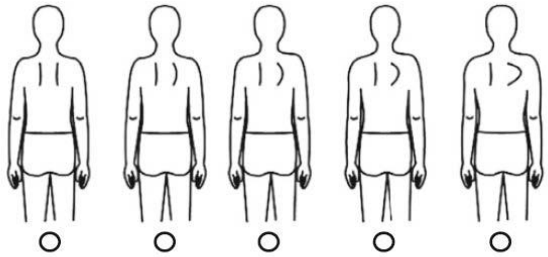

9 **De positie van jouw hoofd**
(Kleur één bolletje in)

Van de zijkant:

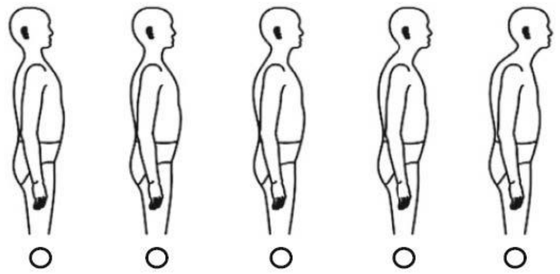

10 **De bolling van jouw rug**
(Kleur één bolletje in)

Van de zijkant:

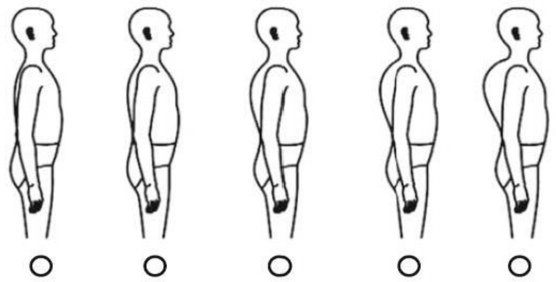

Instructies:

Geef aan of deze uitspraken op dit moment voor jou waar of niet waar zijn:

	**Niet waar**	**Klein beetje waar**	**Beetje waar**	**Redelijk waar**	**Helemaal waar**
**11. Ik wil dat mijn lichaam (links-rechts) meer gelijk is**	○	○	○	○	○
**12. Ik wil dat mijn schouders meer gelijk zijn**	○	○	○	○	○
**13. Ik wil dat mijn heupen meer gelijk zijn**	○	○	○	○	○
**14. Ik wil dat mijn taille meer gelijk is**	○	○	○	○	○

Scoresleutel vragenlijst over het uiterlijk bij scoliose

	**Uiterlijk domein (Appearance)**<br><br>*Let op: maximaal 2 vragen onbeantwoord*	**Verwachtingen domein (Expectations)**<br><br>*Let op: maximaal 1 vraag onbeantwoord*
**Vraag 1**<br>*Scoring: 1 (links) tot 5 (rechts)*		
**Vraag 2**<br>*Scoring: 1 (links) tot 5 (rechts)*		
**Vraag 3**<br>*Scoring: 1 (links) tot 5 (rechts)*		
**Vraag 4**<br>*Scoring: 1 (links) tot 5 (rechts)*		
**Vraag 5**<br>*Scoring: 1 (links) tot 5 (rechts)*		
**Vraag 6**<br>*Scoring: 1 (links) tot 5 (rechts)*		
**Vraag 7**<br>*Scoring: 1 (links) tot 5 (rechts)*		
**Vraag 8**<br>*Scoring: 1 (links) tot 5 (rechts)*		
**Vraag 9**<br>*Scoring: 1 (links) tot 5 (rechts)*		
**Vraag 10**<br>*Scoring: 1 (links) tot 5 (rechts)*		
**Vraag 11**<br>*Scoring: 1 (niet waar) tot 5 (helemaal waar)*		
**Vraag 12**<br>*Scoring: 1 (niet waar) tot 5 (helemaal waar)*		
**Vraag 13**<br>*Scoring: 1 (niet waar) tot 5 (helemaal waar)*		
**Vraag 14**<br>*Scoring: 1 (niet waar) tot 5 (helemaal waar)*		
**Domein score**<br>*Uiterlijk: som scores vraag 1 t/m 10*<br>*Verwachtingen: som scores vraag 11 t/m 14*	**(max. 50)**<br><br>Indien 1 of 2 vragen onbeantwoord, gebruik de volgende formule:<br>*((Domein score/[5·aantal beantwoorde vragen])·50)*	**(max. 20)**<br><br>Indien 1 vraag onbeantwoord, gebruik de volgende formule:<br>*((Domein score/[5·aantal beantwoorde vragen])·20)*
**Totaal score SAQ**<br>*Uiterlijk domein score + verwachtingen domein score*		
